# Metagenomic Analysis of the Dynamic Changes in the Gut Microbiome of the Participants of the MARS-500 Experiment, Simulating Long Term Space Flight

**Published:** 2013

**Authors:** A.V. Mardanov, M.M. Babykin, A.V. Beletsky, A.I. Grigoriev, V.V. Zinchenko, V.V. Kadnikov, M.P. Kirpichnikov, A.M. Mazur, A.V. Nedoluzhko, N.D. Novikova, E.B. Prokhortchouk, N.V. Ravin, K.G. Skryabin, S.V. Shestakov

**Affiliations:** Centre “Bioengineering”, Russian Academy of Sciences, prosp. 60-let Oktyabria, 7/1, Moscow, Russia, 117312.; Biological Faculty, Lomonosov Moscow State University, Leninskie gory, 1/12, Moscow, Russia, 119991; Russian Federation State Research Center Institute of Biomedical Problems RAS (IBMP)

**Keywords:** metagenomics, intestinal microbiota, stressful influences, enterotypes

## Abstract

A metagenomic analysis of the dynamic changes of the composition of the intestinal microbiome of five participants of the MARS-500 experiment was performed. DNA samples were isolated from the feces of the participants taken just before the experiment, upon 14, 30, 210, 363 and 510 days of isolation in the experimental module, and two weeks upon completion of the experiment. The taxonomic composition of the microbiome was analyzed by pyrosequencing of 16S rRNA gene fragments. Both the taxonomic and functional gene content of the microbiome of one participant were analyzed by whole metagenome sequencing using the SOLiD technique. Each participant had a specific microbiome that could be assigned to one of three recognized enterotypes. Two participants had enterotype I microbiomes characterized by the prevalence of *Bacteroides, *while the microbiomes of two others, assigned to type II, were dominated by *Prevotella*. One participant had a microbiome of mixed type. It was found that (1) changes in the taxonimic composition of the microbiomes occurred in the course of the experiment, but the enterotypes remained the same; (2) significant changes in the compositions of the microbiomes occurred just 14-30 days after the beginning of the experiment, presumably indicating the influence of stress factors in the first stage of the experiment; (3) a tendency toward a reversion of the microbiomes to their initial composition was observed two weeks after the end of the experiment, but complete recovery was not achieved. The metagenomic analysis of the microbiome of one of the participants showed that in spite of variations in the taxonomic compositions of microbiomes, the “functional” genetic composition was much more stable for most of the functional gene categories. Probably in the course of the experiment the taxonomic composition of the gut microbiome was adaptively changed to reflect the individual response to the experimental conditions. A new, balanced taxonomic composition of the microbiome was formed to ensure a stable gene content of the community as a whole without negative consequences for the health of the participants.

## INTRODUCTION

Metagenomic studies of the human microbiome conducted within the framework of large-scale international research programs [1-3] are aimed at shedding light on the role microorganisms play in human life, developing diagnostic techniques, and preventing and treating various diseases. The taxonomic and genetic composition of the microbiota inhabiting the intestines is one of the criteria used to assess human health [4-6]. The intestinal ecosystem is dominated by five phyla of bacteria accounting for over 95% of the entire microbiota; however, the proportion of taxa at the genus and species level is specific to each person [2, 7, 8]. The latter is attributed to the genetic characteristics of every individual, the dominant type of nutrition, and the specifics of the interactions between microbes in a holistic ecosystem. Meanwhile, every “healthy” individual is characterized by their own balanced and constant metagenomic composition [8-10], which can vary significantly in the presence of various diseases [5, 7, 11, 12] or due to the impact of medicinal products [7, 13, 14]. The interrelation between the condition of the resident microbiota and the type of nutrition [15-17], psychophysiological, and neurohumoral factors [18-20] has been identified. Stressful physical and emotional overloads affect the composition of the microbiota. Deviations from the usual lifestyle (e.g. long trips) can lead to an imbalance in the ratio of various taxa in the microbiota [11], and they are often accompanied by painful symptoms (diarrhea, constipation, etc.). The conditions inherent in space flights may impose both physical and psychological stresses on astronauts [21, 22], influencing the functioning of their intestinal microbiota [23-25].

The “MARS-500” experiment was conducted at the Institute of Biomedical Problems of the Russian Academy of Sciences. It involved the simulation of some of the conditions of a long interplanetary flight. The participants were put into an isolated module for 510 days in order to investigate the possible influence of the conditions of “space flight” on their physiological and psychological states. The composition of the intestinal microbiota of five subjects was assessed during one of the biomedical tests (in the course of the “MARS-500” experiment their feces were periodically sampled). DNA preparations isolated from feces were used for sequencing, with subsequent determination of the taxonomic and genetic composition of the microbiota.

The study established that a prolonged stay in an isolated module leads to changes in the composition of the microbiota. The dynamics of the changes were specific to each participant. Adaptive restructuring of the intestinal ecosystem apparently occurred, reflecting the individual response of each participant to the influence of experimental conditions (psycho-emotional stress, change in type of nutrition, use of probiotics, etc.). These conditions had no significant negative impact on the health of the participants, as evidenced by the results of medical and biological monitoring of each of the participants’ condition [26, 27].

## EXPERIMENTAL

**Collection of samples for the metagenomic analysis**

Citizens of four countries (Russia, Italy, France, China) aged 28 to 38 selected for inclusion in the crew subject to approval on the basis of the results of medical and psychological testing participated in the “MARS-500” experiment. In the course of the experiment, their feces were sampled at point zero (immediately prior to entering the isolation module), then after 14, 30, 210, 363, 510 days of stay in the module and 2 weeks after exiting the module (524 days). The samples of feces were stored at –80°C; DNA preparations were isolated using the QIAamp DNA stool Mini Kit (Qiagen, Germany), commonly used for the analysis of microbiota in feces. The quality of the DNA preparations was evaluated by agarose gel electrophoresis. It should be noted that the method used for DNA isolation can lead to an underestimation of the proportion of Actinobacteria and overestimation of the proportion of Bacteroidetes [28]; however, the comparative metagenomic analysis at different stages of the experiment is substantiated, as an identical approach was applied to all samples.

**Amplification and pyrosequencing of fragments of 16S ribosomal RNA genes**

In order to perform the PCR amplification of the fragment of the 16S rRN A gene comprising the variable V3–V5 regions barcoded “universal,” the primers PRK341F (5’-CCT ACGGGRBGCASCAG) and PRK806R (5’-GGACT ACYVGGGTATCT AAT) were used. PCR was performed in a volume of 50 μl containing 2.5 units of GoTaq-DNA polymerase (Promega), 0.2 mM MgCl_2_, 0.1 μM of each of the deoxyribonucleoside triphosphates, and 1 μM of each primer and 0.1 μg of the metagenomic DNA. The reaction was carried out using the Eppendorf Mastercycler amplifier (Eppendorf, Germany) according to the following schedule: initial denaturation for 2 min at 96°C, followed by 30 cycles (96°C – 40 s, 58°C – 40 s, 72°C – 1 min), then followed by final elongation for 10 min at 72°C. PCR fragments were purified by agarose gel electrophoresis. The samples were prepared for pyrosequencing according to the standard methodology (excluding the step of DNA fragmentation) using the GS Rapid Library Prep Kit. The GS Titanium LV emPCR Kit (Lib-L) v2 was used for emulsion PCR ; pyrosequencing on the GS FLX (Roche) was performed according to the Titanium protocol using the GS Titanium Sequencing Kit XLR70. Reads exceeding 350 nucleotides in length were selected for further analysis. Thus, 549,668 independent sequences of fragments of the 16S rRN A gene were obtained. They were aligned and filtered using the Mothur software package [29] (version 1.23.1). Chimeric sequences were removed using Chimera.uchime [30], which is part of the Mothur package. Reads that did not pass the filtration process amounted to up to 10% of the total number in different samples. Taxonomic classification of the reads that passed the filtration process was performed using the Wang *et al*. method [31] implemented in the RDP Classifier program. Analysis of the results of the re-sequencing of the four DNA preparations obtained from participants № 1 and № 5 at different stages of the experiment demonstrated that the differences between the parallel samples (with respect to the ratio of fractions of the major taxa) did not account for more than 3% of the total microbial community. This demonstrates the methodological appropriateness of the results shown in the diagrams.

**Sequencing of metagenomes using SOLiD technology**

Libraries of fragments of metagenomic DNA samples were prepared according to the standard methodology using the SOLiD Fragment Library Construction Kit. The sizes of the libraries were measured using the Agilent BioAnalyzer DNA1000 kit. The length of the fragments varied from 183 to 254 bp. Emulsion PCR was performed according to the standard protocols recommended by Applied Biosystems Company using the EZ Bead System. Determination of DNA nucleotide sequences was carried out using 50 bp reads on the SOLiD 4.0 sequencing machine (Applied Biosystems). The volume of sequencing after the filtration with respect to the quality of a reading ranged from 1.8 to 3.4 billion bp per sample. After filtration the reads were collected and assembled into contigs using a parallel version of Abyss 1.2.5 [32]. The search for genes in the contigs and their functional and taxonomic classification were performed on the MG-RAST server (http://metagenomics.anl.gov/) for automatic annotation and analysis of the metagenomic data. This program predicts genes in the contigs on the basis of FragGeneScan [33] and then conducts a search for their homologues [34] in its own M5NR database using BLAT, which integrates multiple databases – GenBank, KEGG, COG, The SEE D [35], and UniProt [36]. During the taxonomic classification each gene was assigned to a family of the closest homologue from the GenBank. Genes containing matches in the KEGG database were assigned several KEGG categories corresponding to different levels of the hierarchy.

The MG-RAST functional and taxonomic classification does not consider a multiplicity of gene readings, and the analysis results were corrected with allowance for the coverage. The nucleotide coverage of the predicted genes was determined by mapping the individual reads onto the assembled contigs using the Bowtie program [37].

**Table 1:** Enterotypes of the microbiota of the participants at point zero of the experiment

Taxonomic affiliation	Participant, №				
	1	2	3	4	5
Firmicutes					
Lachnospiraceae	8.63	12.33	7.59	19.11	15.07
Negativicutes	2.33	4.88	10.35	2.75	7.18
Ruminococcaceae	3.06	19.99	13.66	20.41	5.45
Others	0.6	2.98	3.47	5.60	1.31
Bacteroidetes					
Prevotellaceae	75.25	< 0.01	35.78	9.63	0.03
Rikenellaceae	0.56	1.62	2.77	1.82	1.72
Porphyromonadaceae	0.58	1.23	2.87	5.34	1.31
Bacteroidaceae	2.57	53.36	17.16	28.92	63.82
Others	0.96	0.26	4.62	5.60	0.83
Minor groups					
Proteobacteria	5.09	0.72	1.14	0.15	2.50
Actinobacteria	0.08	0.07	0.02	< 0.01	0.04
Fusobacteriaceae	< 0.01	2.06	< 0.01	< 0.01	< 0.37
Verrucomicrobia	< 0.01	< 0.01	0.31	0.07	< 0.01
Other microorganisms	0.28	0.5	0.27	0.61	0.36
__VOID__					
Number of reads prior to filtration	5450	4883	6253	4929	7882
Number of reads after filtration	5321	4567	5886	4610	7545
Enterotype	II	I	II	III	I

Note. Proportion (%) of the determined 16S rRNA sequences assigned to the respective taxonomic groups.

## RESULTS AND DISCUSSION

**Taxonomic composition of the intestinal microbiome on the basis of the results of pyrosequencing of fragments of the 16S rRNA genes**

Metagenomics methods using 16S rRN A as a marker revealed more than 40 genera of bacteria in the intestinal microbiota of the participants in the “MARS-500” experiment, the majority belonging to the four phyla: Bacteroidetes, Firmicutes, Proteobacteria and Actinobacteria, which is in agreement with data regarding the composition of the intestinal microbiota in healthy adults [38, 39]. Representatives of certain other phyla, including Fusobacteria, Verrucomicrobia and Synergistia, were also identified. Methanogenic archaea (*Methanobrevibacter *genus) were detected in two participants.

A comparative taxonomic analysis of the microbiota in fecal samples obtained from five participants at point zero of the experiment (prior to entering the isolated module) revealed significant individual differences between the participants with respect to the composition of the microbiota. The results obtained (*Table 1*) allowed to determine the belonging of the microbiota to specific enterotypes according to the classification proposed in 2011 [40, 41]. Specific clusters of microbes with the predominance of a particular taxon are designated as enterotypes. These clusters control the food chains in the microbial community and the interaction of the latter with the host characterized by individual genotypic characteristics.

Participants № 1 and № 3 were characterized by enterotype II dominated by *Prevotella, *combined with Firmicutes *Faecalibacterium*, *Coprococcus*,* Blautia*. Minor groups of *Akkermansia *(Verrucomicrobia) and β-proteobacteria were detected in the intestinal microbiota of participant № 3, while participant № 1 was characterized by a high proportion of γ-Proteobacteria.

The microbiota of participants № 2 and № 5 belongs to enterotype I with the predominance of *Bacteroides* in a cluster with *Parabacteroides*, *Faecalibacterium *and certain groups of Ruminococcaceae and Lachnospiraceae.* Fusobacteria *was also detected in these participants. One of the features of the composition of the microbiota of participant № 5 is a relatively high content of β-Proteobacteria, as well as the prevalence of the *Phascolarctobacterium* genus amongst Negativicutes, while the *Dialister *genus is predominant in participant № 2.

A different picture of the taxonomic composition of the microbial community was found in participant № 4. At point zero of the experiment no pronounced predominance of phylogroups determining enterotypes I and II was identified. A high proportion of Ruminococcaceae (including unclassified phylotypes), Lachnospiraceae, as well as Paraprevotella, was found instead. Amongst Negativicutes the genus *Dialister *was predominant as in participant № 2. The microbiota of participant № 4 was characterized by the presence of archaea *Methanobrevibacter*. Thus, the intestinal microbial community of this participant was different and could belong to a mixed type close to enterotype III [41]. Such a mixed composition could be viewed from the perspective of the notions regarding the gradient of microbiome composition as opposed to the concept of discrete enterotypes [42, 43].

**Fig. 1:**
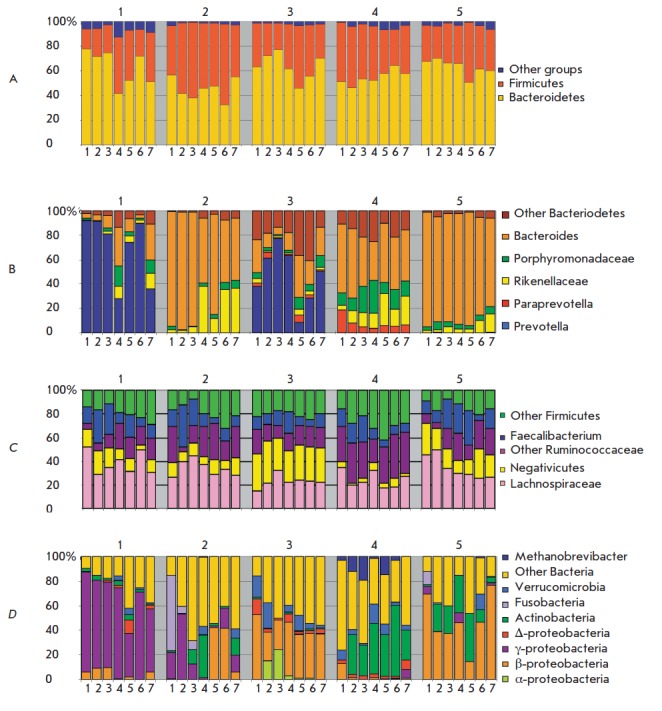
Dynamic changes in the gut microbiome of participants of the MARS-500 experiment. (A) – main groups of microorganisms, (B) – microorganisms of phylum Bacteroidetes, (С) – microorganisms of the phylum Firmicutes, (D) – minor groups. Fraction of sequences assigned to a particular taxonomic group is shown in vertical axis (%), horizontal axis shows the sample codes (1 – 0 days, 2 – 14 days, 3 – 30 days, 4 – 210 days, 5 – 363 days, 6 – 510 days, and 7 – 524 days). Identification numbers of participants are shown above

The results of the metagenomic studies demonstrated that prolonged stay in the isolated module exerted influence on the taxonomic composition of the microbiota of each of the participants (*Fig. 1*). The dynamics of these changes had an individual character reflecting the differences in the initial composition of the microbial communities and the different reactions of the participants to the influence of the conditions/factors of the experiment. As can be seen from *Fig. 1A–D*, a single unidirectional trend in the changes in microbiota composition was absent in the participants from the beginning to the completion of the experiment. The variability of the changes appears to be associated with differences in the conditions at different stages of the experiment. This applies to the administration of the probiotic *Enterococcus faecium *(in the form of tablets during the first 180 days) and Eubikor and Vitaflor during the last months, change in diet, the performance of special types of tasks by certain members of the crew associated with the exit from the main module to the simulated surface of Mars (after 210 days but before sampling after 363 day). All participants received identical probiotics and prebiotics during a single period. Participants № 2, № 3, and № 5 exited the module to the simulated surface of Mars wearing spacesuits.

The individual nature of the response of each participant is reflected in the data regarding the dynamics of the changes in the microbiota at the genus and species level and such indicators as the ratios of the major phyla, Firmicutes (F), and Bacteroidetes (B). With respect to microbiota, the ratio F/B changed significantly in participants № 1, № 2, and № 3 and in participants № 4 and № 5 it remained relatively stable throughout the entire experiment (*Fig. 1A*). The F/B ratio of participant № 1 significantly increased only to the 210^th^ day of stay in the module, while an increase in this index was observed after 2 weeks in participant № 2. However, after 210 days it began to decline. On the contrary during the first month the F/B ratio decreased in participant № 3 and then increased once again. Several studies have shown that abrupt changes in the ratio of Firmicutes/Bacteroidetes occur in the presence of certain gastrointestinal [13, 44] and other diseases [45, 46]. However, symptoms of such diseases were not observed in any of the participants of the “MARS-500” experiment during their stay in the module.

**Fig. 2:**
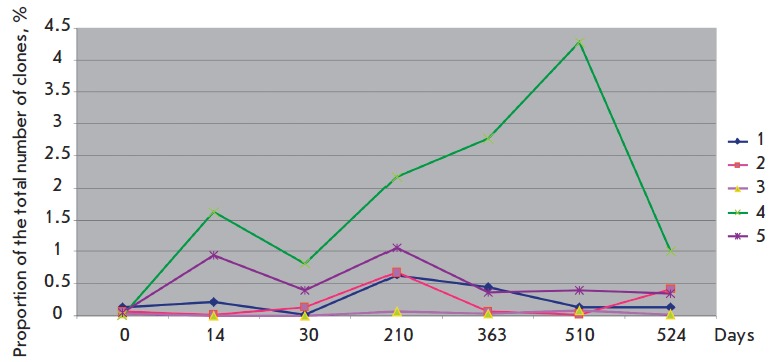
Dynamic changes in the relative abundances of Actinobacteria in the gut microbiome of participants of the MARS-500 experiment

Throughout the entire experiment, there were no changes in the basic enterotype, although the fractional content of individual taxa was significantly altered in the microbiota. The proportion of unidentified bacteria, representatives of Firmicutes (*Fig. 1C*), and Proteobacteria (*Fig. 1D*) increased in participant № 1 from the 210th day, while high levels of *Prevotella*, *Faecalibacterium* and *Coprococcus *remained (*Fig. 1B*). In participant № 2 the loss of Fusobacteria was discovered already on the 14th day of the experiment (*Fig. 1D*) and fluctuations in the relative abundance of bacteria from the genus *Bacteroides *defining enterotype II were detected (*Fig. 1B*). During the first weeks, a slight increase in the proportion of *Faecalibacterium *(with a subsequent decline) and a decrease in the proportion of* Roseburia *with an increase in the minor species of *Alistripes* (*Rikkenellaceae*) and representatives of Lachnospiraceae was recorded. An insignificant decrease in the proportion of *Bacteroides *during the first weeks with an accompanying increase in the relative content of Prevotellaceae (*Fig. 1B*) and the proportion of γ-Proteobacteria, as well as *Megamonas *(Negativicutes) and unclassified groups of bacteria, were detected in the microbiota of participant № 3. The history of the changes in the composition of the majority of species and genera of the intestinal microbiota of participant № 4 showed no significant fluctuations (*Fig. 1*). However, an increase in the relative content of *Faecalibacterium prausnitzii *(*Fig. 1C*) during the first weeks of the experiment and *Roseburia *on the 210th day, as well as an increase in the proportion of Actinobacteria, was clearly identified (*Fig. 2*). No significant changes in the microbiota composition (except for Proteobacteria) in participant № 5 (*Fig. 1A*) were detected. However, a trend towards a decrease in the proportion of *Bacteroides* towards the completion of the stay in the module was identified (*Fig. 1B*).

**Fig. 3:**
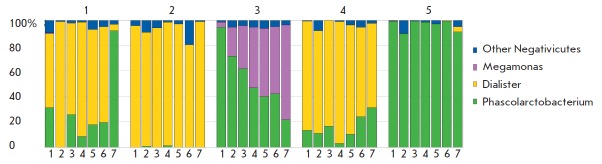
Dynamic changes in the relative abundances of Veillonellaceae in the gut microbiome of participants of the MARS-500 experiment. Fraction of sequences assigned to a particular taxonomic group is shown in vertical axis (%), horizontal axis shows the sample codes (1 – 0 days, 2 – 14 days, 3 – 30 days, 4 – 210 days, 5 – 363 days, 6 – 510 days, and 7 - 524 days). Identification numbers of participants are shown above

The detailed comparative analysis of the microbiota profiles revealed certain patterns in the dynamics of the content of Actinobacteria and Negativicutes. The Actinobacteria content was minimal in the initial samples of the microbiota of all participants, which could potentially be attributed to the peculiarities of the methods of DNA extraction and/or use of primers, which were ineffective for obtaining fragments of bifidobacteria 16S rRN A. As can be seen from *Fig. 2*, the samples obtained at different stages of the experiment demonstrated an increased relative content of Actinobacteria, especially in the microbiota of participant № 4. This increase in the proportion of Actinobacteria can probably be attributed to the intake of probiotics, as it could stimulate the growth of bifidobacteria. It is possible, however, that this increase in the proportion of Actinobacteria was determined by their more active dissociation from the surface of the epithelium at the sites where colonization occurred. If the composition of Negativicutes at the genus level did not change significantly during the experiment for participants № 2, № 4 and № 5, the microbiota of participant № 3 revealed a consistent replacement of bacteria from the genus *Phascolarctobacter *with bacteria from the genus *Megamonas *(*Fig. 3*) without restoration of the initial composition of Negativicutes 2 weeks after exiting the module.

The following trends could be noted during the analysis of the dynamics of the changes in the composition of the intestinal microbiota occurring in the course of the experiment. First, the impact of conditions/factors of the experiment was observed during the first weeks, although to varying degrees for different participants. It is believed that the rapid changes were caused by the initial psychological and emotional reaction to the unusual stressful conditions of containment in the isolated module. Second, there was a tendency toward partial recovery of the initial composition of the microbiota with respect to individual groups of taxa after the completion of the experiment. However, none of the participants demonstrated complete recovery of their initial composition 2 weeks after exiting the module. It is known that the use of antibiotics that cause drastic changes in the composition of the intestinal microbial community is followed by initiation of recovery in the initial composition after discontinuation of the medicinal product [14]. However, even partial recovery of the composition of the indigenous microbiota requires prolonged periods of time [47].

**Determination of the gene composition of the microbiota of participant № 2**

The results of the analysis of the taxonomic composition of the microbiome with respect to the sequences of 16S rRN A genes presented above did not provide direct information regarding the set of functional genes in the microbial metagenome. Therefore, we determined the gene composition of the samples of the microbiota of participant № 2. This participant demonstrated noticeable changes in the taxonomic composition of microorganisms in the course of the experiment.

During the analysis of the results of the sequencing of samples of metagenomic DNA according to the SOLiD technique, one must consider the following: 1) the average length of the contigs for different points did not exceed 200 nucleotides; i.e., it was significantly smaller than the average size of a bacterial gene, and 2) the Bacteroidetes present in the microbiome were represented mainly by the *Bacteroides *genus (complete genomic sequences of many species from this genus had been determined). The bacteria of the phylum Firmicutes were phylogenetically more diverse. Therefore, the taxonomic identification of contigs belonging to Bacteroidetes was relatively more complete, whilst many contigs *de facto *belonging to Firmicutes could not be classified due to the lack of close homologues in the databases. This led to an underestimation of the proportion of Firmicutes in the metagenome compared to the results of the 16S rRN A analysis. Nevertheless, the dynamics of the changes in the ratio between Bacteroidetes and Firmicutes remained the same.

**Fig. 4:**
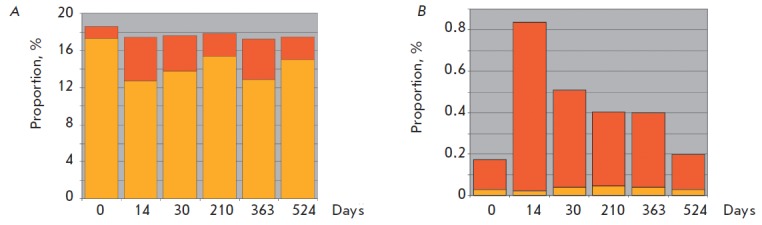
Dynamic changes in the fractions of genes assigned to KEGG functional categories “Carbohydrates metabolism” (A) and “Cell motility” (B) in the gut microbiome of participant 2. Fraction of genes assigned to a particular category is shown in vertical axis (%), horizontal axis shows the sample codes (1 – 0 days, 2 – 14 days, 3 – 30 days, 4 – 210 days, 5 – 363 days, 6 – 510 days and 7 - 524 days). Fractions of genes taxonomically assigned to Bacteroidetes are shown in yellow, those assigned to *Firmicutes*, in orange

Quantitative representation of the genes of certain functional categories in the metagenome (according to KEGG classification, [34]) and their assignment to various taxonomic groups of bacteria were characterized. In general, significant changes in the microbiota of participant № 2 in the course of the experiment were absent with respect to the major functional categories of genes. Thus, the KEGG category “Carbohydrates metabolism,” one of the most important for the functioning of the intestinal microbiota, at various stages of the experiment was represented by 16.7 to 18.6% of the identified genes (*Fig. 4A*). However, the relative contribution of various taxa of Bacteroidetes and Firmicutes changed in a significantly wider range as evidenced by the results of the “taxonomic” classification of these genes and the data obtained on the basis of the taxonomic analysis with respect to 16S rRN A. We can assume that the process of restructuring of the taxonomic composition of the microbiome involved the replacement of genes in various representatives of Bacteroidetes and Firmicutes that determine the metabolism of carbohydrates, although the overall proportion of this functional category in the metagenome remained almost unaltered.

A different picture was obtained during the analysis of the dynamics of changes in the proportion of genes of the KEGG category “Cell motility” responsible for cellular motility (*Fig. 4B*). The majority of the genes in this category was assigned to Firmicutes and, accordingly, their proportion in the metagenome varied with changes in the relative content of Firmicutes and, perhaps, Proteobacteria in the community. These data are consistent with a small number of genes that determine cellular motility in the sequenced genomes of members of the genus *Bacteroides*. Cellular motility in Firmicutes and Proteobacteria is controlled by a large number of genes [48]. Flagella not only provide mobility but also perform sensory functions and are involved in intercellular communication in ecosystems [49]. Perhaps the “demand” for cellular motility determined the increase in the proportion of certain phylotypes of Firmicutes in the restructuring of the microbial community in the course of the experiment.

It can be assumed that during the “MARS-500” experiment, adaptive restructuring of the microbial community occurred in response to the stressful condition of prolonged isolation. Likewise, the formation of a new and balanced taxonomic composition of the microbiota occurred, providing maintenance of the normal functioning of the genetic and metabolic networks in the intestinal microbial community and in the system of interactions between the microbiota and the host organism. This adaptive transition to a new combination of taxa with preservation of the optimal gene composition in the entire community can be achieved through redundancy of the majority of categories of genes and functional interchangeability of bacterial phylotypes from different taxonomic groups. An exchange of genes by horizontal gene transfer [48], which is possible with the involvement of viruses, mobile elements and conjugative plasmids that are common in many microbes inhabiting the intestine, can be one of the mechanisms of such interchangeability.

## CONCLUSIONS

The results of the metagenomic analysis of the intestinal microbiota of the participants of the “MARS-500” experiment simulating some of the conditions of long interplanetary flights suggest that containment in an isolated module is associated with the microbiota undergoing substantial changes in the composition of the microbial community. These changes were specific to each of the participants, which is attributable to the differences in the initial composition of the microbiota and the different nature of the responses to the influence of the experimental conditions depending on the genetic, physiological, and biochemical characteristics of each participant.

The factors affecting the taxonomic composition of the microbiota include the psychological stress attributed to the change in lifestyle, the switch to a different type of nutrition, and the use of probiotics. Monitoring of the dynamics of the changes in the microbiota demonstrated that (1) significant changes in the taxonomic composition began to appear during the initial stages of the experiment; (2) changes in the enterotypes in the individual taxonomic groups did not occur despite the large variability: i.e., the basic composition of the intestinal ecosystem remained unchanged; and (3) 2 weeks after exiting the module a tendency toward a return to the initial composition of the microbiota was observed; however, none of the participants demonstrated complete restoration of the initial composition of their microbial community. Perhaps, a two-week period of “rehabilitation” was simply insufficient for such recovery to occur.

As during the experiment, none of the participants demonstrated symptoms of diseases associated with considerable changes in the composition of the microbiota [6, 11, 12]: it can be assumed that restructuring of the taxonomic composition occurred in their intestinal ecosystems, reflecting their individual responses to the conditions of the experiment and a new balanced community was formed. This hypothesis is supported by the data on the analysis of the gene composition of the microbiota of participant № 2. The gene composition of the metagenome of the intestinal microbiota of this participant experienced little change throughout the experiment, which could be attributed to a compensatory substitution of certain species/strains with others able to perform functions related to ensuring “normal” interaction between the microbial community and the host organism.

Thus, it can be concluded that the powerful stressful condition of prolonged containment in an isolated module had no “dramatic” effect on the state of the intestinal microbiota and did not lead to significant negative consequences for the health of the participants of the experiment. Obviously, isolation during long space flights is just one of the stress factors that have the potential to affect astronauts. With proper selection and training of a crew, this factor could be rendered moot. Zero gravity, radiation, and certain specific working conditions in a spaceship may be more significant. These factors, which are difficult to reproduce during an experiment on Earth, can increase the likelihood of functional disorders in the gastrointestinal tract, the immune and other systems, potentially leading to the development of dysbiosis manifested as significant changes in the genetic and taxonomic composition of the intestinal microbiota. The data obtained during this experiment regarding the changes in the composition of the intestinal microbiota of the participants of the “MARS-500” experiment should be considered with respect to the possibilities of using methods of metagenomic analysis of the microbiota as one of the approaches to testing the state of health of the participants of real space flights and candidates for performing work under extreme conditions associated with powerful stressful factors.
